# The New Anæsthetic, Bichloride of Methylene

**Published:** 1868-04

**Authors:** 


### The New Anæsthetic, Bichloride of Methylene.

Dr. Richardson claims for this substance the following characters:

1—It is an effective general anæsthetic, producing as deep insensibility as chloroform.

2—In action it is rather more rapid than chloroform, but to develop effects more of it is required, in the proportion of six parts to four.

3—It produces a less prolonged second degree of narcotism than other anæsthetics.

4—When its effects are fully developed, the narcotism is very prolonged, and is reproduced with great ease.

5—Its influence on the nervous centres is uniform, and it creates little if any disturbance or break of action between the respirating and circulating functions.

6—Its final escape from the organism is rapid, so that the symptoms of recovery are sudden.

7—In some cases it produces vomiting.

8—When it kills, it destroys by equally paralyzing the respirating and circulating mechanisms.

9—It interferes less with the muscular irritability than perhaps any other anæsthetic.

10—It combines with ether and with chloroform in all proportions.

Dr. R. appends to his elaborate lecture, descriptive of this agent, the following account of its trial on the human subject. The whole paper is most valuable and interesting, but entirely too lengthy for transfer to our columns:

Since the lecture above written was delivered I have been enabled to test the action of the bichloride of methylene on the human adult subject in five long and severe operations. Four of these were cases of ovariotomy performed by Mr. Spencer Wells, and the period of narcotism in each case averaged from thirty-five to forty-five minutes. In all the cases, except one where Weiss's inhaler was used, the anæsthetic was administered from a simple mouth-piece made of a layer of parchment paper stretched over a light frame of wood, and lined on the inner surface with lint. The quantity of bichloride of methylene used in each case averaged a little more than a fluid drachm, each five minutes, two drachms being first used. In all the cases the administration has been safe, and the recovery from the effects of the narcotic good. I noticed especially, that in each case the transition from the first to the third degree of narcotism was very brief; that when the anæsthesia, which was complete in an average of five minutes, was well established, it was easily prolonged for six, and even seven minutes, without re-administering the vapor, and when recovery began to show itself, very brief re-administration quickly reproduced the insensibility. In one case after the operation, the patient continued twenty-seven minutes in unbroken sleep, and then awoke with entire consciousness.

It happened that one of the ladies to whom I gave the anæsthetic had once been under chloroform, Dr. Snow having been the administrator. This lady was therefore able to compare the effects of the two agents, and she gave her verdict strongly in favor of the bichloride. She said it caused no sense of suffocation, no ringing sounds in the head, no nausea, and no after depressing effect whatever, as chloroform in her case did; but it allowed her to drop into sleep precisely as in natural sleep, and to wake with all her senses aroused as after natural sleep.

On the whole, the results of practice, in so far as they go, have fully realized my expectations. Only two adverse points, and those minor, occur to me. In one case—after operation for vesico-vaginal fistula—the patient, who recovered from the anæsthesia without any nausea, had, I learn, ten hours afterwards, a bilious vomit. It would be unfair to put this against the anæsthetic, but the fact should be stated. In another case, the anæsthesia having been carried very deeply, the tongue of the patient was retracted into the throat, and after pulling it forward, there was a free secretion of saliva, followed by an eructation and about a dessert-spoonful of fluid from the stomach. This might have been excited by the irritation communicated to the throat in pulling up the tongue, or it might have been from the anæsthetic. In all the cases the recovery from the anæsthetic was in every sense satisfactory.

It is already stated in the lecture that bichloride of methylene mixes with ether. I have tried this mixture in experiments as an anæsthetic, using equal parts of ether and bichloride. The compound anæsthetics quickly, and the action is characteristically modified. The ether materially shortens the duration of the insensibility, there is less freedom of respiration, and more excitement. On the whole, I do not at this moment see any advantage in the mixture over the simple bichloride. I leave the point open, however, for future study.

I leave the Bichloride of Methylene with the profession for its observation and experience. I have proved the agent, by experiment on the lower animals, to be a good general anæsthetic. I have inhaled it myself with safety, and I have administered it to the human subject with success in the extremest operations for which general anæsthesia is demanded. Here, as an individual inquirer, I come back into the ranks and rejoin the rest of my brethren as an observer. Having no other ambition than that of being a physician in the widest sense; having even a painful aversion to specialty, and having no desire to press my subject unduly, I have produced this lecture as a contribution to pure science, and nothing more; holding myself as free as any one else to condemn, improve, or approve, as future knowledge, framed and squared and fitted by wisdom, shall determine. When twenty thousand persons shall have slept away pain under the influence of "Chloromethyl," as Mr. Spencer Wells has tersely named the Bichloride of Methylene, and those of them who have slept too deeply shall be counted as fewer than ten, an advance over chloroform will have been proved, but not sooner, nor with less of that tribulation through which we must ever attain to the good that is great and persistently beneficent.—*Medical Times and Gazette.*

